# Supplementary material for: Plug-and-Display Photo-Switchable Systems on Plant Virus Nanoparticles
Source: BioTech (Basel). 2022 Oct 21;11(4):49. doi: 10.3390/biotech11040049 (PMC9589989; doi:10.3390/biotech11040049)
Supplement: Supplementary file 1 [file biotech-11-00049-s001.zip › biotech-1908978-supplementary.pdf]

Supplementary materials: Plug-and-display photo-switchable systems on plant virus nanoparticles

Louisa Kauth, Eva Miriam Buhl, Julian Luka, Karolin Richter, Ulrich Commandeur and Christina Dickmeis

1. Plasmid construction

All cloning procedures were carried out using standard protocols (Sambrook & Russell, 2001). All restriction enzymes were purchased from New England Biolabs (Ipswich, MA, USA). Pfu polymerase (Promega, Madison, WI, USA) was used for PCR cloning and Taq polymerase (Institute of Molecular Biotechnology, RWTH Aachen University, Germany) was used for check-PCR with the primers listed in Error! Reference source not found.. Cloning vectors were dephosphorylated with calf intestinal phosphatase (New England Biolabs) before ligation with T4 DNA ligase (Promega) overnight at 16 °C. Gibson Assembly Master Mix was used according to the manufacturer's recommendations (New England Biolabs) without vector dephosphorylation. *E. coli* DH5α cells were transformed with ligation or Gibson assembly reaction products and colonies were selected overnight at 37 °C on lysogeny broth agar plates supplemented with 100 µg/ml ampicillin. Plasmids were isolated from overnight cultures and sequenced by Eurofins Genomics (Ebersberg, Germany) using the Mix2seq Kit.

2. LOVTRAP

The Zdk1 sequence was originally derived from pTriEx-NTOM20-mVenus-Zdk1, a gift from Klaus Hahn (Addgene plasmid #81010; http://n2t.net/addgene:81010; RRID:Addgene_81010). The sequence was amplified by PCR (primers GA_Zdk1-Duet-fw and GA_Zdk1-mCherry-rev) along with mCherry (primers GA_mCherry-Zdk1-fw and GA-mCherry-Duet-rev), and the fragments were inserted into the BamHI/NotI site of pETDuet-1 (Novagen/Merck, Darmstadt, Germany) by Gibson assembly.

The LOV2 sequence, originally obtained from pTriEx-NTOM20-LOV2, a gift from Klaus Hahn (Addgene plasmid #81009; http://n2t.net/addgene:81009; RRID:Addgene_81009) was amplified by PCR using primers GA_LOV2-SC-fw and GA_LOV2-GEX-rev. A GSGSG linker was added between the SC and LOV2 sequences by PCR using primers GA_SpyCatcher-Duet-fw and GA_SpyCatcher-LOV2-rev. Both PCR fragments were inserted into the BamHI/SalI sites of pGEX-5x-1 by Gibson assembly. The GST and SC sequences were fused to the LOV2 N-terminus because a free C-terminus is required to bind Zdk1 (Wang et al., 2016). The cloned constructs were named pETDuet-his6-Zdk1-mCherry and pGEX-SC-LOV2.

3. BphP1/QPAS1

The QPAS1 sequence from pQP-iRIS was a gift from Vladislav Verkhusha (Addgene plasmid #102584; http://n2t.net/addgene:102584; RRID:Addgene_102584) and was amplified by PCR using primers GA_QPAS1-SC-fw and GA_QPAS1-GEX-rev. A GSGSG linker was added between the SC and QPAS1 sequence by PCR using primers GA_SpyCatcher-Duet-fw and GA_SC-QPAS1-rev. Both fragments were inserted into the BamHI/SalI sites of pGEX-5x-1 by Gibson assembly. The restriction sites SpeI and NotI were added to the mCherry sequence by PCR using primers SpeI-mCherry and mCherry-NotI-His$_6$. Restriction and ligation into the SpeI/NotI sites of pET22b-BphP1-mVenus-His$_6$ resulted in the replacement of mVenus with mCherry. The BphP1-mVenus sequence was derived from pQP-iRIS. The cloned constructs were named pGEX-SC-QPAS1 and pET22b-BphP1-mCherry-His$_6$.

4. Dronpa145N

The SpeI and XhoI sites were added to the Dronpa145N sequence (pcDNA3-mNeptune2-N was a gift from Michael Lin, Addgene plasmid #41645; http://n2t.net/addgene:41645; RRID:Addgene_41645) by PCR using primers SpeI-Dronpa145N and Dronpa145N-Stop-XhoI. The PCR fragment was cloned into vector pCR 2.1 (Thermo Fisher Scientific) and, after restriction, ligated into the SpeI/SalI sites of pETDuet, which already carried a (G4S)$_3$ linker and the mCherry or SC sequence (Institute of Molecular

Biotechnology, RWTH Aachen University). The cloned constructs were named pETDuet-His6-SC-G4S-Dronpa145N and pETDuet-His6-mCherry-G4S-Dronpa145N. The His6 tag, SC and mCherry were fused to the N-terminus of Dronpa145N based on previous studies (Zhou et al., 2012).

All constructs were cloned and sequenced successfully.

Table S1. Oligonucleotides used in this study.

Primer	Sequence (5′→3′)
**pETDuet-His6-Zdk1-mCherry**	
GA_Zdk1-Duet-fw	ACCATCATCACCACAGCCAGGTGGATAACAAATTCAATAAAGAAAAGACGC
GA_Zdk1-mCherry-rev	CCTTGCTCACTTTTGGGGCCTGGGCATC
GA_mCherry-Zdk1-fw	GGCCCCAAAAGTGAGCAAGGGCGAGGAG
GA-mCherry-Duet-rev	GTTCGACTTAAGCATTATGCCTTGTACAGCTCGTCCATGC
**pGEX-SC-LOV2**	
GA_SpyCatcher-Duet-fw	GATCTGATCGAAGGTCGTGGGGATTACGATATCCCGACGACG
GA_SpyCatcher-LOV2-rev	TGCGGCCCACACCACTGCCGCTACCGATGTGGGCATCCCCCTTG
GA_LOV2-SC-fw_neu	TGCCCACATCGGTAGCGGCAGTGGTTTGGCTACTACACTTGAAC
GA_LOV2-GEX-rev	TCACGATGCGGCCGCTCGAGTTAAAGTTCTTTTGCCGC
**pGEX-SC-QPAS1**	
GA_SpyCatcher-Duet-fw	GATCTGATCGAAGGTCGTGGGGATTACGATATCCCGACGACG
GA_SC-QPAS1-rev	TGTTCTTGCCACCACTGCCGCTACCGATGTGGGCATCCCCCTTG
GA_QPAS1-SC-fw	TGCCCACATCGGTAGCGGCAGTGGTGGCAAGAACATGCAGGCG
GA_QPAS1-GEX-rev	TCACGATGCGGCCGCTCGAGTTAGATCGCGGGAGTCGTG
**pET22b-BphP1-mCherry-His6**	
SpeI-mCherry	AAAACTAGTGTGAGCAAGGGCGAGGAGGA
mCherry-NotI-His6	ATGGTGATGAGCGGCCGCCTTGTACAGCTCGTCCATG

pETDuet-His$_6$-SC-G4S-Dronpa145N<br>pETDuet-His$_6$-mCherry-G4S-Dronpa145N	
SpeI-Dronpa145N	ACTAGTATGAGCGTGATCAAGCCCGAC
Dronpa145N-Stop-XhoI	CTCGAGTTACTTGGCCTGCCTGGGCAGCTC

Transmission electron microscopy analysis

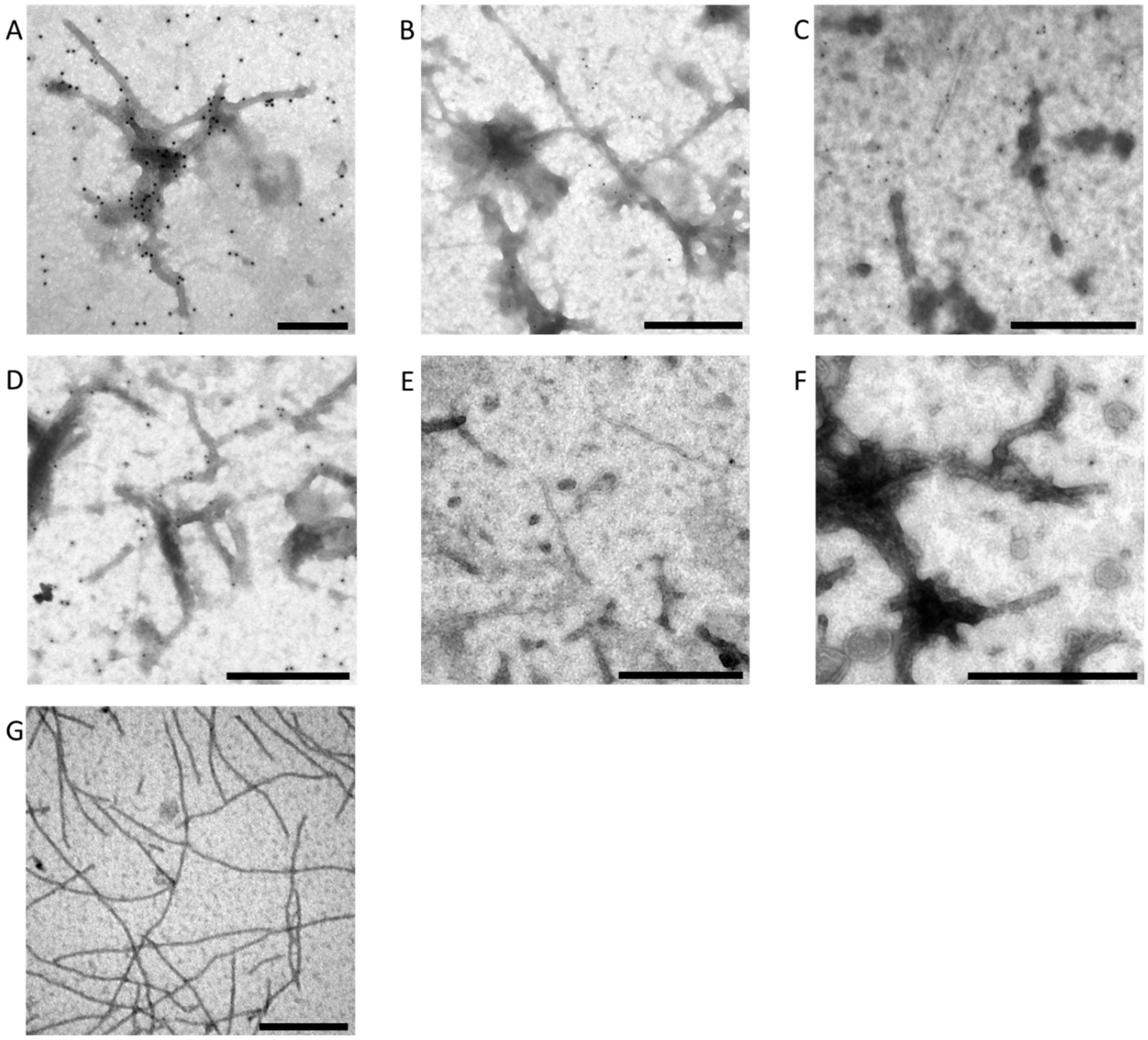

Figure S1. Transmission electron micrographs showing controls of the LOVTRAP system on PVX particles. **A)** Purified PVX-ST/GST-SC-LOV2 particles were mixed with His$_6$-Zdk1-mCherry and incubated in the dark. Particles were attached to the grids and decorated with anti-mCherry/GAM-12 nm gold conjugate. **B)** Purified PVX-ST/GST-SC-LOV2 particles were attached to the grids in the dark and decorated with an anti-His$_6$/GAM-12 nm gold conjugate. **C)** Purified PVX-ST/GST-SC-LOV2 particles were attached to the grids in the dark and decorated with an anti-mCherry/GAM-12 nm gold conjugate. **D)** Purified PVX-ST/GST-SC-LOV2 particles were mixed with BphP1-mCherry-His$_6$ and incubated in the dark. Particles were attached to the grids under blue light and decorated with an anti-mCherry/GAM-12 nm gold conjugate. **E)** Purified PVX-ST/GST-SC-LOV2 particles were attached to the grids under blue light and decorated with an anti-His$_6$/GAM-12 nm gold conjugate. **F)** Purified PVX-ST/GST-

SC-LOV2 particles were attached to the grids under blue light and decorated with an anti-mCherry/GAM-12 nm gold conjugate. **G)** Wild-type PVX particles were irradiated with blue light and attached to the grids. Scale bars = 250 nm (A, D-G) and 500 nm (B, C).

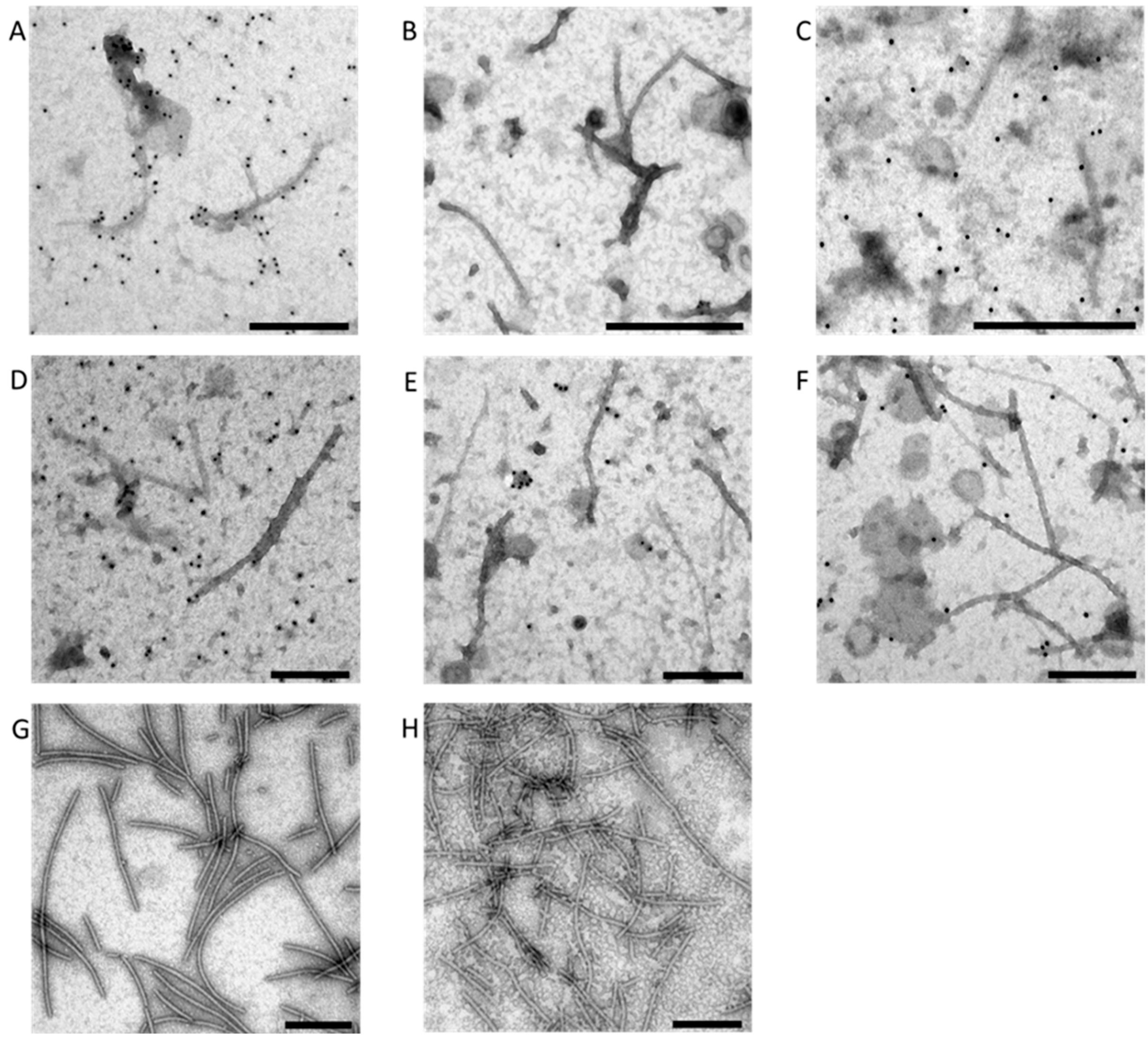

Figure S2. Transmission electron micrographs showing controls of the BphP1/QPAS1 system on PVX particles. **A)** Purified PVX-ST/GST-SC-QPAS1 particles were mixed with BphP1-mCherry-His_6 and irradiated at 780 nm. Particles were attached to the grids and decorated with an anti-mCherry/GAM-12 nm gold conjugate. **B)** Purified PVX-ST/GST-SC-QPAS1 particles were irradiated at 780 nm. Particles were attached to the grids and decorated with an anti-His_6/GAM-12 nm gold conjugate. **C)** Purified PVX-ST/GST-SC-QPAS1 particles were irradiated at 780 nm. Particles were attached to the grids and decorated with an anti-mCherry/GAM-12 nm gold conjugate. **D)** Purified PVX-ST/GST-SC-QPAS1 particles were mixed with BphP1-mCherry-His_6 and irradiated at 630 nm or incubated in the dark. Particles were attached to the grids and decorated with an anti-mCherry/GAM-12 nm gold conjugate. **E)** Purified PVX-ST/GST-SC-QPAS1 particles were irradiated at 630 nm or incubated in the dark. Particles were attached to the grids and decorated with an anti-His_6/GAM-12 nm gold conjugate. **F)** Purified PVX-ST/GST-SC-QPAS1 particles were irradiated at 630 nm or incubated in the dark. Particles were attached to the grids and decorated with an anti-mCherry/GAM-12 nm gold conjugate. **G)** Wild-type PVX particles were irradiated at 780 nm and attached to the grids. **H)** Wild-type PVX virus particles were irradiated at 630 nm and attached to the grids. Scale bars = 250 nm (A, D-H) and 500 nm (B, C).

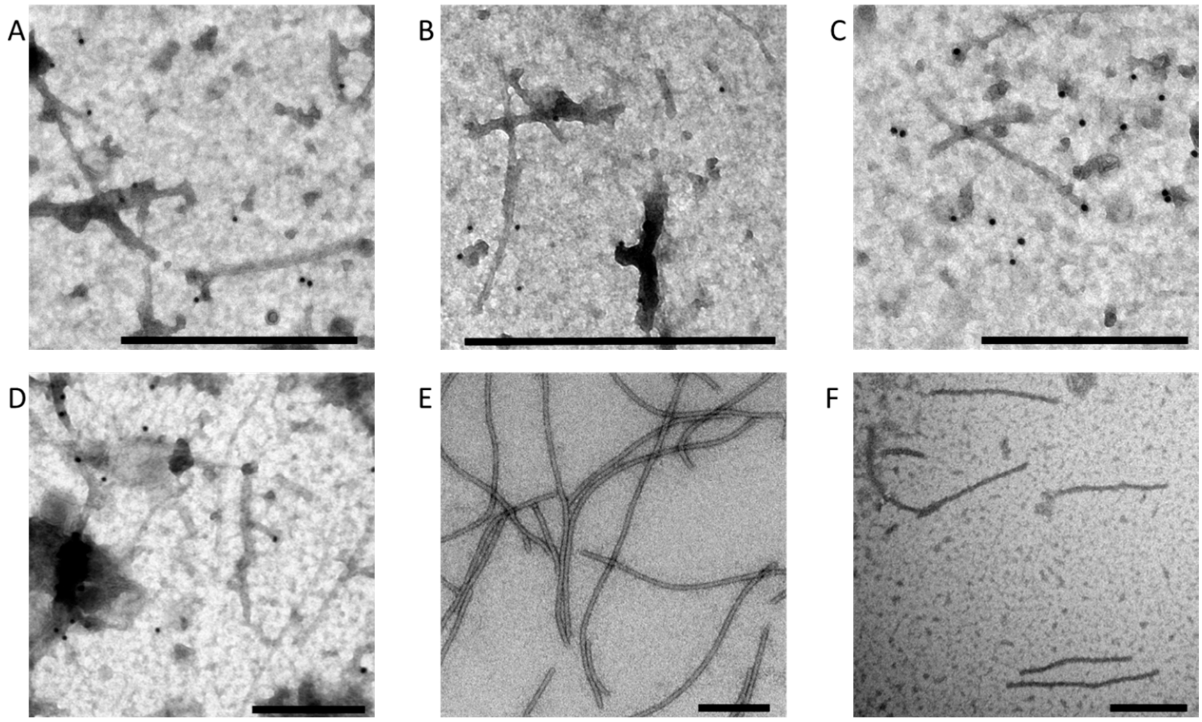

Figure S3. Transmission electron micrographs showing controls of the Dronpa145N system on PVX particles. A) Purified PVX-ST/His_6-SC-G4S-Dronpa145N particles were mixed with His_6-mCh-G4S-Dronpa145N protein and irradiated with violet light (405 nm). Particles were attached to the grids and decorated with an anti-mCherry/GAM-12 nm gold conjugate. **B)** Purified PVX-ST/His_6-SC-G4S-Dronpa145N and His_6-mCh-G4S-Dronpa145N proteins were irradiated with cyan light (505 nm) before mixing. The particles were attached to grids and decorated with an anti-mCherry/GAM-12 nm gold conjugate. **C)** PVX-ST/His_6-SC-G4S-Dronpa145N particles were mixed with His_6-mCherry-G4S-Dronpa145N in the dark and decorated with an anti-mCherry/GAM-12 nm gold conjugate. **D)** PVX-ST/His_6-SC-G4S-Dronpa145N particles in the dark were decorated with an anti-mCherry/GAM-12 nm gold conjugate**. E)** Wild-type PVX particles were irradiated with violet light (405 nm) and attached to the grids. **F)** Wild-type PVX particles were irradiated with cyan light (505 nm) and attached to the grids. Scale bars = 500 nm (A, C), 1 μm (B) and 250 nm (D–F).

Light equipment

Table S2. List of LED-sources and power supplies.

Name	Type	Manufacturer
KPS101	Power supply	Thorlabs, Newton, NJ, USA
LED Strip 5050 60 LED/m Infrared 740nm	740 nm LED Strip	Buyledstrip.com
LED Strip 5050 60 LED/m UV 400nm	400 nm LED Strip	Buyledstrip.com
LED Strip flexible 120 LED/m Blue	470 nm LED-Strip	Buyledstrip.com
LED Strip Red 120 LED/m Waterproof	630 nm LED Strip	Buyledstrip.com
LEDD1B	T-Cube LED driver	Thorlabs, Newton, NJ, USA
M405L4	405 nm high power LED	Thorlabs, Newton, NJ, USA
M455L4	455 nm high power LED	Thorlabs, Newton, NJ, USA
M505L4	505 nm high power LED	Thorlabs, Newton, NJ, USA
M780L3	780 nm high power LED	Thorlabs, Newton, NJ, USA
Power supply 40 Watt Meanwell	Power supply	Buyledstrip.com
